# Liquid–Solid Triboelectric Nanogenerator‐Based DNA Barcode Detection Biosensor for Species Identification

**DOI:** 10.1002/advs.202408718

**Published:** 2024-12-04

**Authors:** Wenlong Ma, Jiawei Li, Xiaolin Qu, Shao‘e Sun, Yanan Zhou, Yitong Liu, Peng Wang, Zhongli Sha

**Affiliations:** ^1^ Key Laboratory of Advanced Marine Materials Key Laboratory of Marine Environmental Corrosion and Bio‐fouling Institute of Oceanology Chinese Academy of Sciences Qingdao 266071 China; ^2^ Institute of Marine Corrosion Protection Guangxi Key Laboratory of Marine Environmental Science Guangxi Academy of Marine Sciences Guangxi Academy of Sciences Nanning 530007 China; ^3^ Department of Marine Organism Taxonomy & Phylogeny Institute of Oceanology Chinese Academy of Sciences Qingdao 266071 China; ^4^ Laoshan Laboratory Qingdao 266237 China; ^5^ Shandong Province Key Laboratory of Experimental Marine Biology Institute of Oceanology Chinese Academy of Sciences Qingdao 266071 China

**Keywords:** biosensor, DNA barcode detection, species identification, triboelectric nanogenerator

## Abstract

DNA barcode detection method is widely applied for species identification, which is imperative to evaluate the effect of human economic activities on the biodiversity of ecosystem. However, the wide utilization of existing detection biosensors is limited by bulky and expensive instruments, such as Raman spectroscopy and electrochemical station. Herein, a liquid–solid triboelectric nanogenerator (TENG)‐based DNA barcode detection biosensor is proposed, which consists of water flow, fluid channel, and PDMS film attached by specifically designed capture probe. Through sequentially combining capture probe, targeted DNA barcode, and signal probe with Au nanoparticles (NPs), the surface charge density of friction layer of TENG decreases under the effect of AuNPs, verified by the density functional theory (DFT) method. Consequently, the peak value of output current spike signal for targeted DNA is smaller than that for other DNA, which is the working mechanism of the present TENG‐based biosensor. Such biosensor successfully recognizes *Alvinocaris muricola* among different types of *Alvinocarididae* shrimps, and its low limit detection can reach 1×10^−12^ m. The present work provides a paradigm‐shift way to develop an inexpensive and accurate technique to detect DNA barcode for species identification, and paves a novel way for the application of liquid–solid TENG.

## Graphical abstract

This work develops a liquid–solid TENG‐based DNA barcode detection biosensor for species identification, based on the effect of surface conditions on the output current signal. This work provides a novel low‐cost and portable detection method to investigate the change of species in certain environments, helping to evaluate the effect of human economic activities on the ecosystem, such as hydrothermal vents.

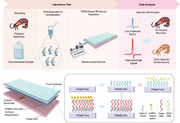

## 1 Introduction

With large‐scale human economic activities develop to extreme environments, such as deep‐sea mining, the biodiversity may be significantly threatened by pollution, leading to the great loss of genetic resources.^[^ ^1^, ^2^, ^3^, ^4^, ^5^ ^]^ Therefore, there demands investigations on the change of species inhabiting various environments, providing a scientific guideline for future human activities.^[^ ^6^ ^]^ During the process of investigating the potential loss of species diversities, species identification techniques are inevitably applied. Conventional species identification requires taxonomic specialists to distinguish the morphological characters of physical creatures,^[^ ^7^, ^8^ ^]^ making it time‐consuming, and easy to false identification. Thus, DNA barcoding has been proposed as a novel molecular tool for species identification,^[^ ^9^, ^10^, ^11^ ^]^ due to that it is more objective and accurate, less time‐consuming.

To rapidly and accurately detect the DNA barcode for species identification, various kinds of biosensors have been proposed, based on the different transduction mechanisms.^[^ ^12^, ^13^, ^14^ ^]^ Recently, an emerging biosensor based on TENG, which is able to easily convert tiny mechanical stimuli into electricity,^[^ ^15^, ^16^, ^17^ ^]^ has attracted massive attention. Compared with conventional biosensors, TENG‐based biosensors^[^ ^18^, ^19^, ^20^, ^21^, ^22^, ^23^, ^24^ ^]^ not only eliminate the need for bulky and expensive detection instruments, such as Raman and electrochemical station,^[^ ^25^, ^26^ ^]^ but also can generate signals without a power supply. The above unique characteristics make TENG‐based biosensors portable, easy to handle, low cost, and energy consumption, therefore, a series of them have been proposed for different working fields. Until now, a majority of previous TENG‐based biosensors have been designed, based on the contact separation mode TENG. Specifically, the adsorbed molecules may change the surface charge of the friction layer of TENG, and the output electrical signal changes with the specific targeted molecules,^[^ ^23^, ^24^, ^27^ ^]^ which can be applied for detection. However, repetitive contact separation working mode may destroy molecules absorbed on the friction layer by the mechanical force^[^ ^28^, ^29^, ^30^ ^]^ or corona discharge,^[^ ^31^, ^32^, ^33^ ^]^ severely deteriorating the sensitivity and lifetime of the TENG‐based biosensor. Consequently, it is imperative to develop a novel durable TENG‐based DNA barcode detection sensor.

Compared with solid‐solid TENG, liquid–solid TENG attracts a great amount of attention and is consequently designed for various types of sensors,^[^ ^34^, ^35^, ^36^, ^37^ ^]^ due to its low friction and long lifetime. However, to the best of our knowledge, liquid–solid TENG has not been applied as a DNA barcode detection biosensor. In the present study, a liquid–solid TENG‐based DNA barcode detection sensor, consisting of water flow, fluid channel and PDMS film with capture probe and signal probe, is consequently developed to distinguish *Alvinocaris muricola*. And the combined methodology of COMSOL simulation and DFT is applied to investigate how the signal probe affects the output current signal of TENG‐based biosensor. A series of experiments have been sequentially conducted to optimize the structure and working conditions of TENG. The optimized TENG‐based biosensor is utilized to measure different DNA barcode of various species of shrimps, and *Alvinocaris muricola* is successfully distinguished. The present study develops a novel DNA barcode detection biosensor for species identification, contributing to environmental conservation during human economic activities.

## 2 Results and Discussion

### 2.1 Design and Fabrication of TENG‐Based Biosensor for Hydrothermal Vent Ecosystem

**Figure 1:**
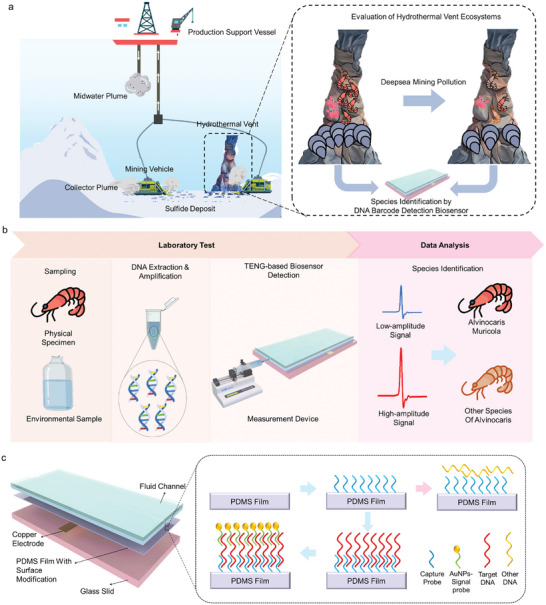
The concept application, structure, and working mechanism of the liquid–solid TENG‐based DNA barcode detection biosensor. a) The concept application of TENG‐based biosensor for species identification of hydrothermal vent ecosystem under the effect of deep‐sea mining. b) The working process of species identification by the liquid–solid TENG‐based biosensor. c) The structure of liquid–solid TENG‐based biosensor with the magnified view of PDMS film with different surface conditions for different DNA. The blue arrow represents the successful combination of the capture probe, target DNA and AuNPs‐signal probe. The pink arrow represents no combination between the capture probe, other DNA and AuNPs‐signal probe.

As shown in **Figure** **1**a, the hydrothermal vent, formed by seawater circulation through hot volcanic rocks, is a valuable deep‐sea mining site, due to their high deposits of rare earth minerals, including gold, silver, and copper. Besides, these vents host unique ecosystem of hundreds of new species. Studying these new species can not only help trace the origin of life on Earth, but also enrich genetic resources and inspire novel medical applications. However, the operation of mining vehicles generates current plumes, intense noise, and light pollution, which may impair the ecosystem of hydrothermal vents, leading to changes or loss of inhabiting species. Therefore, it is crucial to detect typical species of hydrothermal vent ecosystems, to ascertain the potential effect of deep‐sea mining on biodiversity.

As an endemic typical species inhabiting hydrothermal vent, *Alvinocaris muricola* is chosen to explain how to apply DNA barcode detection biosensor to evaluate the change of biodiversity, shown in Figure 1b. First, the physical specimen or environmental seawater sample is collected from a hydrothermal vent by an underwater vehicle. Then the DNA barcode is selected and amplified, followed by being detected by the self‐designed liquid–solid TENG‐based DNA barcode detection biosensor. According to the working principle of such biosensor in Section 2.3, the peak value of the electrical current signal can be applied to distinguish *Alvinocaris muricola* from other *Alvinocarididae* shrimps, due to the evidently discrepant surface conditions of TENG friction layer for different types of *Alvinocarididae* shrimps.

To detect the DNA barcode (sequence shown in Table S1, Supporting Information), a DNA barcode detection rig is proposed, which consists of a syringe pump and a liquid–solid TENG‐based biosensor (Figure 1b). As the core composition of the detection rig, the biosensor is composed of a glass slid, copper electrode, polydimethylsiloxane (PDMS) film, and fluid channel, as shown in Figure 1c. To specifically detect the DNA barcode of *Alvinocaris muricola*, the PDMS surface is initially functionalized by 3‐aminopropyltriethoxysilane (APTES), followed by introducing amino groups. The above chemically modified PDMS film surface is conjugated by the capture probe, specifically designed for the DNA barcode of *Alvinocaris muricola*. The PDMS film is sequentially submerged in DNA barcode solution, which is previously collected from physical specimens or environmental samples. When the collected DNA barcode is the target DNA, the target DNA is anchored on the capture probe. Additionally, another DNA, which is designed to specifically combine with the rest of the target DNA, is incorporated by AuNPs. The incorporated DNA, called by the AuNPs‐signal probe, is applied to ascertain the target DNA again. Due to the existence of AuNPs, the surface condition of PDMS film changes, leading to a low‐amplitude of liquid–solid TENG output current signal. As for other DNA barcodes, they cannot combine with the capture probe and AuNPs‐signal probe, the surface condition of PDMS film is different from that of PDMS film for target DNA, leading to a discrepant current signal output. Therefore, the *Alvinocaris muricola* can be identified by the liquid–solid TENG‐based biosensor current signal to investigate the effect of deep‐sea mining on the biodiversity of the hydrothermal vent.

### 2.2 Characterization of Fabrication of Capture Probe and Signal Probe

**Figure 2:**
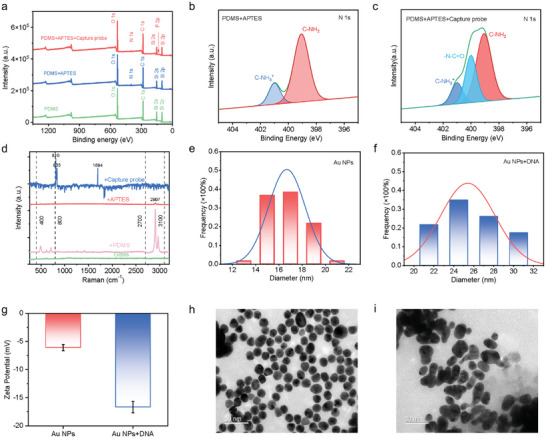
Characterization of synthetic components of liquid–solid TENG‐based biosensor. a) XPS spectra of the PDMS surface with different chemical modifications (green line represents unmodified PDMS, blue line represents PDMS with APTES modification, and red line represents PDMS with additional modification of capture probe). High‐resolution N1s spectra of the PDMS film with chemical modifications of b) APTES and c) additional capture probe. d) Raman spectra of PDMS with different chemical modifications. Size distribution of e) AuNPs and f) combination between AuNPs and DNA (AuNPS+DNA). g) Zeta potential of AuNPs and AuNPS+DNA. TEM image of h) AuNPs and i) AuNPS+DNA.

According to the design of the biosensor in the above section, the successful fabrication of the capture probe and AuNPs‐signal probe (synthesis process can be seen in Notes S1 and S2, Supporting Information) is essential to accurately detect the DNA barcode of *Alvinocaris muricola*. So, it is necessary to conduct a series of characterization tests to verify whether both probes are successfully fabricated. **Figure** **2**a–c illustrates the XPS analysis results of the PDMS film surface during the chemical modification process. As shown in Figure 2a, a new N peak at 400 eV appears after the first surface modification, suggesting the PDMS film has been successfully functionalized by APTES. Additionally, the N1s spectrum of XPS analysis results in Figure 2b illustrates that two peaks at 399.5 and 401.4 eV, which are associated with C─NH_2_ and C─NH_3_^+^, are detected, respectively. Contrarily, no such peak is found in the N1s spectrum of PDMS (Figure S1, Supporting Information). Besides, the contact angle of water on the PDMS surface dropped from 115° to 102° (Figure S2a,b, Supporting Information), suggesting the existence of the hydrophilic amino group on the PDMS surface. The above characterization results verify the successful modification of the PDMS surface with APTES.

On the basis of the PDMS surface modified with APTES, the capture probe is sequentially conjugated, needing characterization as well. As shown in Figure 2a, a P peak at 133 eV, which is the essential element of DNA, appears after the conjugation of the capture probe. Moreover, a new peak at 400.2 eV is found in the N1s spectrum of XPS analysis results (Figure 2c), suggesting the existence of ‐N─C═O. The XPS results illustrate that the capture probe is successfully conjugated by the PDMS with APTES. During the fabrication process of the biosensor, Raman spectroscopy is applied to analyze chemical bonds, as shown in Figure 2d. The peaks of Si─O─Si, Si─CH_3_, and SiC appear in the range from 400 to 800 cm^−1^, and an evident peak of C─H exists in the range from 2700 to 3100 cm^−1^, suggesting the existence of PDMS film on the glass slid. After PDMS film is chemically modified by ATPES, an evident C─NH_2_ peak exists at 2900 cm^−1^. Sequentially, evident peaks of DNA are found at 815, 828, and 1044 cm^−1^, suggesting that the capture probe is successfully conjugated on the PDMS film. In conclusion, as the core composition of the biosensor, the PDMS film with the capture probe is successfully fabricated.

Despite the capture probe, the signal probe, consisting of the DNA probe and AuNPs, is another core composition of the biosensor, enabling the liquid–solid TENG output current signal to distinguish the DNA barcode of *Alvinocaris muricola*. Therefore, the characterization of the AuNPs is needed. First, the UV–vis test results (Figure S3, Supporting Information) illustrate that no absorption peak is found for the HAuCl_4_ solution, however, an evident adsorption peak at 521 nm for the mixed solution between HAuCl_4_ and Na_3_C_6_H_5_O_7_. The results suggest that the AuNPs are successfully synthesized. As shown in Figure 2e, the size distribution analysis results of the TEM image (Figure 2h) of AuNPs indicate that the mean particle size is ≈16 nm. After the combination between DNA and AuNPs, the same characterization tests are conducted to analyze as well. The UV–vis adsorption peak of AuNPs increases to 525 nm (Figure S3, Supporting Information), and the mean particle size rises to 24 nm (Figure 2f,i), due to the addition of the DNA. Additionally, due to the electrical negativity of DNA, the combination of DNA leads the Zeta potential of AuNPs to decrease from −7.5 to −17 mV. The above characterization results suggest that the signal probe, consisting of AuNPs and DNA, is successfully synthesized.

### 2.3 Working Principle of TENG‐Based DNA Barcode Detection Biosensor

**Figure 3:**
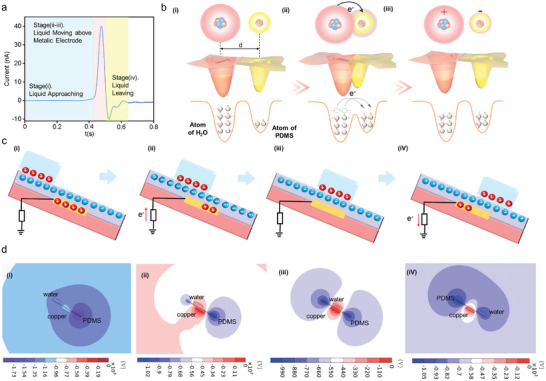
Analysis of the working mechanism of liquid–solid TENG. a) Typical current signal generated by liquid–solid TENG. b) The electron‐cloud‐potential‐well model of charge transfer between PDMS and water. c) Charge distribution and d) electric potential distribution of i–iv) four stages of the working process of liquid–solid TENG.

According to Section 2.1, the liquid–solid TENG with friction layers at different surface conditions is applied to detect the DNA barcode. Therefore, it is imperative to investigate the fundamental working principle of liquid–solid TENG, to better understand the effect of surface modifications on the signal output. **Figure** **3**c shows the working process of the liquid–solid TENG for PDMS film. In stage I, when flowing water contacts with the PDMS film, the water is positively charged and PDMS is negatively charged, due to the electron transfer^[^ ^38^, ^39^ ^]^ (Figure 3b). Specifically, when the electron clouds of water molecules and PDMS overlap, the electrons are transferred from water molecules to PDMS (Figure 3b(ii)). After separation, the PDMS film owns more electrons and water has less, leading to different forms of electrostatic charges (Figure 3b(iii)). With the water flowing to the upside of the copper electrode, the opposite electric double layers are formed, leading to the decrease of the induced charges on the copper electrode.^[^ ^40^, ^41^ ^]^ Therefore, the electrons transfer from the ground to the copper electrode (Figure 3c(ii)). Until the whole area of the copper electrode is covered by flowing water, there is no electron transfer between the ground and the copper electrode (Figure 3c(iii)). Followingly, water continues to flow and the area of the copper electrode covered by water consequently decreases, leading to electrons transfer from the copper electrode to the ground. Eventually, the typical signal of such liquid–solid TENG for PDMS film is characterized by a current spike, as shown in Figure 3a. To demonstrate the above working process of liquid–solid TENG, the electric potential distributions are analyzed by the finite element software COMSOL Multiphysics. As shown in Figure 3d, the electric potential around the copper electrode first increased with the area of copper covered by water. When the area of the copper electrode covered by water decreases, the electric potential reduces. The simulation results in Figure 3d are consistent with the analysis of the working process in Figure 3c, demonstrating the above working mechanism.

**Figure 4:**
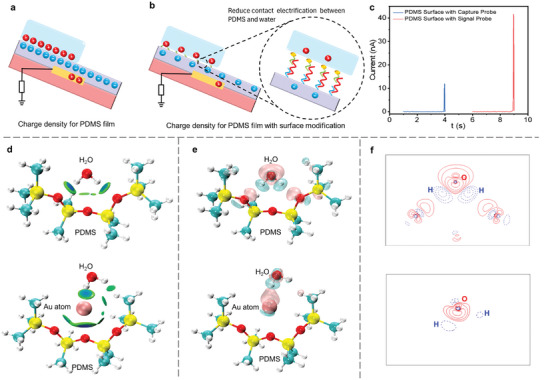
Analysis of surface charge density of liquid–solid TENG with different surface conditions. Surface charge density of water and PDMS film a) without surface modifications and b) with capture probe, DNA barcode, and AuNPs‐signal probe. c) Typical TENG output current signal for PDMS film with different surface conditions. d) IGMH isosurfaces (isovalue = 0.005 a.u.) for water‐PDMS and water‐Au/PDMS. e) 3D charge deformation density isosurfaces for water‐PDMS and water‐Au/PDMS, the red and blue isosurfaces represent the regions in which the electron density is increased and decreased, respectively. f) Contour plot of difference electron density maps, the red and blue curves show positive and negative difference electron densities, respectively.

When the PDMS film of liquid–solid TENG‐based biosensor is anchored by the signal probe (**Figure** **4**b), the surface charge density of water and PDMS film with AuNPs will decrease, compared with TENG‐based biosensor without surface modifications (Figure 4a). Consequently, the induced charge in the copper electrode for TENG with modified PDMS film drops as well, leading to the decrease of the transferred charges between the copper electrode and ground during water flowing process. Eventually, the current signal generated by liquid–solid TENG with PDMS modified by AuNPs signal probes is smaller than that for liquid–solid TENG without any modifications (Figure 4c), suggesting that the current signal can be applied to detect DNA barcode.

To verify the above underlying mechanism, the effect of AuNPs on the surface charge density for liquid–solid TENG was closely examined using DFT calculations. For comparative analysis, two interaction systems were simulated. In the first system, water molecule directly interacted with PDMS molecule. In the second system, water molecules interacted with PDMS with the presence of Au atoms. To understand the nature of non‐covalent interactions in these two systems, the IGMH (Independent gradient model based on Hirshfeld partition) method was employed. The IGMH isosurfaces are depicted in Figure 4d, where blue isosurfaces represent prominent attractive weak interactions such as H‐bonding, red isosurfaces indicate prominent repulsive interactions such as steric effects, and green isosurfaces correspond to van der Waals interactions. The obtained IGMH isosurfaces suggest that, compared to the system with the presence of Au, the attractive interactions are more significant when water molecules directly interact with PDMS. Specifically, the hydrogen atoms of water molecules tend to interact with the oxygen atoms in the PDMS backbone, which is consistent with previous studies.^[^ ^42^ ^]^ In the second system, Au atoms interact with water and PDMS through attractive and van der Waals interactions, and the effective interaction between water and PDMS is reduced. It is worth noting that notable repulsive interactions were not observed in either of the two systems, indicating that the effect of Au on water‐PDMS interaction cannot be simply interpreted as a steric effect.

To further understand the role of Au in the variation of TENG output, the charge deformation density was analyzed. 3D charge deformation density isosurfaces for different systems are presented in Figure 4e. Intuitively, when water molecules come into contact with PDMS, charge transfer from water to PDMS can occur, resulting in the negative charging of PDMS. These DFT computational results are consistent with experimental observations, and the charge‐transfer mechanism can be explained by the overlapping of electron clouds between water molecules and PDMS. During the contact process, the electron‐rich regions of water molecules interact with the electron‐deficient regions of PDMS, facilitating the transfer of electrons from water to PDMS and leading to the accumulation of negative charge on PDMS (red isosurfaces around PDMS). However, with the presence of Au, the transferred electrons are decreased. The charge‐transfer processes were further quantified using charge decomposition analysis. The number of transferred electrons from water to PDMS is 0.04064. However, during the contact between water and Au/PDMS, the number of transferred electrons decreases to 0.036285, indicating that the presence of Au suppresses the charge‐transfer between water and PDMS. This phenomenon can also be observed in the contour plot of difference electron density maps (Figure 4f). Taken together, the DFT calculation results demonstrate that the presence of AuNPs on the PDMS surface can inhibit the transfer of electrons from water to PDMS, providing the basis for quantifying captured DNA based on the variation of TENG output.

### 2.4 Optimization and Demonstration of TENG‐based DNA Barcode Detection Biosensor

**Figure 5:**
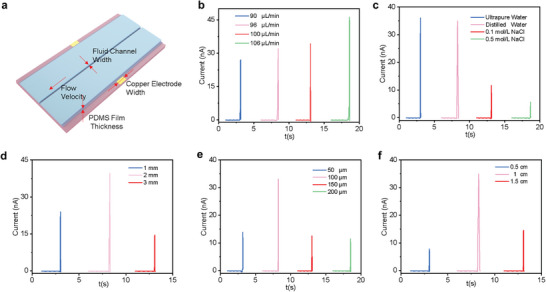
Optimization of working conditions and structure parameters of liquid–solid TENG. a) Schematic of liquid–solid TENG structure and working conditions. TENG output current signal for different b) flow velocities, c) water type, d) fluid channel width, e) PDMS film thickness, and f) copper electrode width.

Based on the working mechanism of the liquid–solid TENG‐based DNA barcode detection biosensor, a higher current signal generated by TENG contributes to better distinguishing the DNA barcode. Therefore, it is imperative to optimize structure parameters and working conditions of liquid–solid TENG (**Figure** **5**a) to improve the output performance. As a significant friction layer, the flow velocity and type of water need to be chosen. Figure 5b illustrates that the peak value of the current spike signal increases from 26.9 nA for 90 µL min^−1^ to 46.3 nA for 106 µL min^−1^, suggesting that a higher flowing water velocity contributes to improving the output performance. The above phenomenon, which may be attributed to a higher water overlapping speed for a larger flowing velocity, is consistent with other investigations about liquid–solid TENG.^[^ ^43^ ^]^ Moreover, the current signal for various kinds of water is obtained as well, as shown in Figure 5c. It can be seen that the peak value of the current signal for ultrapure water and distilled water is significantly higher than saline water with different concentrations of NaCl. Because the high concentration of ions in the saline solution leads to an electrostatic shielding effect on the electric double layer at the solid–liquid interface.^[^ ^44^ ^]^

Besides, the effect of liquid–solid TENG structure parameters on the output performance is also investigated. Figure 5d illustrates that the peak value of the current signal for a 2 mm fluid channel width is higher, compared with the fluid channel width of 1 and 3 mm. It may be caused by a coupling effect of flow velocity and liquid–solid contact area. Specifically, with the increase of fluid channel width, though the liquid–solid contact area increases, the flow velocity decreases. Consequently, there demands above comparison to obtain the best fluid channel width. The PDMS film thickness and copper electrode width may also affect the output performance of liquid–solid TENG through contact electrification and electrostatic induction. Through the comparison in Figure 5e,f, the optimized PDMS film thickness and copper electrode width are 100 µm and 1 mm, respectively.

**Figure 6:**
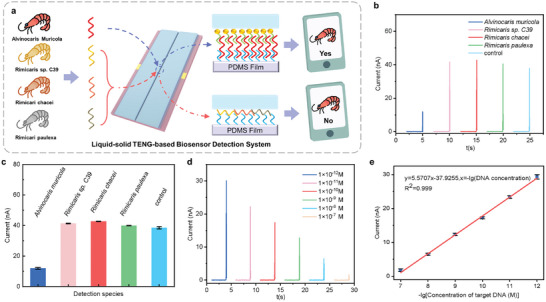
Demonstration of liquid–solid TENG‐based biosensor detection system. a) Schematic of TENG‐based DNA barcode detection system. b) Typical output current signal and c) average peak value of current signal generated by liquid–solid TENG‐based biosensor for various kinds of *Alvinocarididae* shrimps. d) Typical output current signal for *Alvinocaris muricola* DNA barcode with different concentrations ranging from 1×10^−12^ to 1×10^−7^ m. e) Fitted response curve of liquid–solid TENG‐based biosensor at different concentrations.

To demonstrate the specificity of the present liquid–solid TENG‐based DNA barcode detection biosensor, the biosensor is applied to distinguish the *Alvinocaris muricola* among various species of shrimps (**Figure** **6**a), including *Rimicaris sp. C39*, *Rimicarl chacel*, and *Rimicari paulexa* (the DNA sequences are shown in Table S1, Supporting Information). As shown in Figure 6b,c, the average peak value of the current spike generated by liquid–solid TENG‐based biosensor for target DNA is evidently lower than that for non‐target DNA sequences of other species, suggesting that the present biosensor can successfully identify *Alvinocaris muricola* from other shrimp species. Besides, when the target DNA is mixed with DNA sequences of other species of shrimps, the present TENG‐based biosensor can still successfully distinguish the DNA‐barcode of *Alvinocaris muricola* (details can be seen in Note S5, Supporting Information), demonstrating the present biosensor can specifically identify the target DNA regardless of interfering effect of other DNA sequences. Additionally, Figure S13 (Supporting Information) illustrates that peak value of current signal for mismatched target DNA sequences is similar to that for blank group, which is evidently higher than that for target DNA. Therefore, it can be concluded that liquid–solid TENG‐based biosensor can specifically identify the correct target DNA, regardless of the mismatched DNA sequences.

DNA barcodes with different concentrations have been detected. As shown in Figure 6d, the peak value of the current spike signal decreases with the increase of DNA barcode concentration in the range from 1×10^−12^ to 1×10^−7^ m. The liquid–solid TENG‐based biosensor exhibits an excellent linear response to the logarithm of DNA barcode concentration (Figure 6e). And corresponding equation is y = 5.5707x‐37.9255 and R^2^ = 0.999. Therefore, the liquid–solid TENG signal can be successfully applied to distinguish the *Alvinocaris muricola*, and shows promise for application in species identification.

Though the novel liquid–solid TENG‐based biosensor shows great promise for species identification, there still exists potential challenges in large‐scale commercial use in the future. First, the present novel biosensor is still human‐made, and scaling up the production of that inevitably leads to a high fabrication cost. Second, the bottom substrate and flow channel are made from glass, making the present biosensor fragile during the transportation process. Third, the attachment of the capture probe on the PDMS film may not work if the biosensor is kept for about two months. Overall, the liquid–solid TENG‐based biosensor is still in its infancy stage, the development of which still needs to address numerous potential challenges.

## 3 Conclusion

Herein, a liquid–solid TENG‐based DNA barcode detection biosensor is developed for species identification. The friction layer (PDMS film) of liquid–solid TENG is chemically conjugated by the capture probe, enabling to specifically recognize the target DNA barcode of *Alvinocaris muricola*. The other section of the target DNA barcode is sequentially specifically attached by signal probe including AuNPs. Under the effect of AuNPs, the surface charge density of water and PDMS film decreases, which is verified by the COMSOL simulation and DFT methods. The peak value of the output current signal consequently drops, which can be applied to distinguish *Alvinocaris muricola*. Through the comparison of output current signal for various conditions, the optimized flow velocity, water type, fluid channel width, PDMS film thickness, and copper electrode width are 106 µL min^−1^, ultrapure water, 2 mm, 100 µm, and 1 mm, respectively. The optimized biosensor is utilized to detect various kinds of *Alvinocarididae* shrimps, and the *Alvinocaris muricola* is successfully recognized, demonstrating the applicability of the liquid–solid TENG‐based biosensor. And its lower limit detection of DNA can reach 1×10^−12^ m. The present liquid–solid TENG‐based DNA barcode detection biosensor shows promise for species identification, helping to evaluate the effect of human activities on the ecosystem.

## 4 Experimental Section

### Materials and Characterization

As the friction layer of TENG, PDMS was purchased from Corning Inc (USA). During the surface chemical modification process, the materials, including APTES, MES, NHS, and EDC, were from Aladdin (China). All of oligonucleotide chain (Table S1, Supporting Information) were purchased from Sangon Biotechnology Co. Ltd. (Shanghai, China). Despite the materials, a series of characterization were applied in the present research. A programmable electrometer (Keithly Instruments model 6514) was applied to measure the output current signal of liquid–solid TENG. To ascertain the successful synthesis of AuNPs, SEM (Regulus 8100, Hitach), XPS (Escalab 250xi), UV–vis (Hitachi U‐2900 spectrophotometer), Raman (InVia, Renishaw) were utilized. And Malvern Zetasizer Nano ZS90 was used to measure the zeta potential.

### Fabrication of TENG‐Based Biosensor

First, a glass slid (50×50×2 mm) was applied as the substrate, and a copper electrode were attached on the middle of glass slid. Then, PDMS film needed to be spin coated on glass slid with copper electrode, which need preparation of PDMS solution. Mix the PDMS mother liquor, curing agent and ethyl acetate in a mass ratio of 10:1:1, stir at room temperature for 30 min. Then kept it for 1 h until the bubbles disappeared completely. Sequentially, a vacuum coater was applied to spin‐coat the PDMS solution on the glass surface with copper electrodes. After solidification of PDMS solution at room temperature, washed the surface with ethanol and put it in a 50 °C oven for 3 h. Soaked the PDMS in a 1 m KOH solution for 4 h, then washed it twice with deionized water and ethanol, and dried it with nitrogen. So, the PDMS surface hydroxylation modification was completed. Next, treated the PDMS with an APTES solution (5% v/v, dissolved in ethanol) overnight. Then, washed the channel with ethanol, and heated the glass slid with PDMS film at 80 °C for 10 min, filled it with PBS and stored it at 4 °C for later use. The PDMS amination is eventually completed, which was sequentially conjugated with capture probe.

### COMSOL Simulation

COMSOL Multiphysics software is applied to investigate the electric potential distribution during the working process of liquid–solid TENG. The 2‐D numerical model is constructed based on AC/DC modules, electrostatic interfaces, and steady state analysis method.

### DFT Method

In order to investigate the effect of Au on TENG output, density functional theory (DFT) method was adopted to investigate the contact between water and PDMS with/without the presence of Au. The structural optimization was performed by using CP2K package^[^ ^45^ ^]^ (version 2022.1) with DZVP‐MOLOPT‐SR‐GTH basis sets. In addition, the B3LYP functional and mixed GPW basis set with 400 and 50 Ry relative plane‐wave cutoffs were adopted. DFT‐D3 has been included to account for dispersion energy corrections. Based on the optimized molecular topologies, the single‐point energy and wavefunction information were performed in the same B3LYP‐D3 level of theory in CP2K with the def2‐TZVP basis set. The Multiwfn^[^ ^46^ ^]^ program is used to further analyze the atomic interactions of different systems. The interaction structure and calculation results, such as the Independent Gradient Model based on IGMH,^[^ ^47^ ^]^ were visualized and rendered by VMD.^[^ ^48^ ^]^

### Optimization and Demonstration test of TENG‐Based Biosensor

The output current signal of liquid–solid TENG with different working conditions (flowing velocity, water type) and structure parameters (fluid channel width, PDMS film thickness, copper electrode width) was measured, to choose the best liquid–solid TENG. The optimized TENG was applied to detect DNA of *Alvinocaris muricola, Rimicaris chacei, Chorocaris paulexa, Rimicaris sp. C39*, to demonstrate the applicability of the present biosensor. Different concentration of DNA of *Alvinocaris muricola* was measured by the present biosensor to evaluate its accuracy.

## Conflict of Interest

The authors declare no conflict of interest.

## Supporting information



Supporting Information

## Data Availability

The data that support the findings of this study are available from the corresponding author upon reasonable request.
