# Supplementary material for: Liquid–Solid Triboelectric Nanogenerator‐Based DNA Barcode Detection Biosensor for Species Identification
Source: Adv Sci (Weinh). 2024 Dec 4;12(4):2408718. doi: 10.1002/advs.202408718 (PMC11775567; doi:10.1002/advs.202408718)
Supplement: Supplementary file 1 — Supporting Information [file ADVS-12-2408718-s001.docx]

Liquid-solid Triboelectric Nanogenerator-based DNA Barcode Detection Biosensor for Species Identification

*Wenlong Ma, Jiawei Li, Xiaolin Qu, Shao‘e Sun, Yanan Zhou, Yitong Liu, Peng Wang**, *Zhongli Sha**

W. Ma, J. Li, Y. Zhou, Y. Liu, P. Wang

Key Laboratory of Advanced Marine Materials

Key Laboratory of Marine Environmental Corrosion and Bio-fouling

Institute of Oceanology

Chinese Academy of Sciences

Qingdao, 266071, China

*E-mail: wangpeng@qdio.ac.cn

S. Sun, Z. Sha

Department of Marine Organism Taxonomy & Phylogeny

Institute of Oceanology

Chinese Academy of Sciences

Qingdao, 266071, China

*E-mail: shazl@qdio.ac.cn

S. Sun, Z. Sha

Laoshan Laboratory

Qingdao, 266237, China

S. Sun, Z. Sha

Shandong Province Key Laboratory of Experimental Marine Biology

Institute of Oceanology

Chinese Academy of Sciences

Qingdao, 266071, China

**Table S1.** Scheme of synthesized oligonucleotides sequences

| **DNA** | **Sequences (5**′**-3**′**)** |
| --- | --- |
| Target DNA  (*Alvinocaris muricola*) | 5′-CCTGGCAGCAGGAATTGCCCACGCCGGCGCATC  TGTTGACATAGCAATTTTTTCACTTCACCTTGCAGGAGTATCTTCAATCCTAGGCGCCGTCAATTTTATAACCACATGCATTAACATGCGAACAAGA-3′ |
| Reference DNA 1  (*Rimicaris sp. C39*) | 5′-GCAGCAGGCATTGCTCATGCTGGAGCCTCTGTC  GACATAGGGATTTTCTCTCTTCATCTTGCTGGAGTTTCTTCCATCTTAGGAGCCGTAAACTTTATAACAACATGCATCAATATACGAACAAGAGGAA-3′ |
| Reference DNA 2  (*Rimicaris chacei*) | 5′-TGGAAACGATCAAATTTATAATGTTATTGTCACT  GCTCACGCCTTCGTTATAATTTTCTTTATAGTAATACCTATTATAATAGGTGGATTTGGAAATTGACTCATGCCCCTTATACTAGGAGCTCCAGAC-3′ |
| Reference DNA 3 (*Chorocaris paulexa*) | 5′-GACACTATATTTTGTATTCGGAGCATGAGCAGGA  ATAGTAGGAACTGCTCTTAGTCTCCTAATCCGAGCAGAACTAGGTCAACCAGGAAGACTTATCGGAAATGATCAAATTTATAACGTTATTGTCACT-3′ |
| Capture probe | 5′-COOH-GGCGTGGGCAATTCCTGCTGCCAGGTTTT  TTTTTT-3′ |
| Signal probe | 5′-TCTTGTTCGCATGTTAATGCATGTGTTTTTTTTT  T-3′-SH |
| DNA with three mismatches | 5′-GCTGGCAGCAGGATTTGCCCACGCGGGCGCATC  TGTTGACATAGCAATTTTTTCACTTCACCTTGCAGGAGTATCTTCAATCCTAGGCGCCGTCAATTTTATAACCACATGCATTAACATGCGAACAAGA-3′ |
| DNA with five mismatches | 5′-GGTGGCAGCAGGATTTGCCCACGGGGGCGCATC  TGTTGACATAGCAATTTTTTCACTTCACCTTGCAGGAGTATCTTCAATCCTAGGCGCCGTCAATTTTATAACCACATGCATTAACATGCGAACAAGA-3′ |

Table S2. Comparison between TENG-based biosensors with other sensors with different transduction methods.

| Transduction Methods | Linear range | LOD | Ref. |
| --- | --- | --- | --- |
| DPV | 0.01-1.0 nM | 12.0 pM | 1 |
| CV | 20.0 pM-100.0 nM | 2.4 pM | 2 |
| EIS | 2.0 fM-2.0pM | 1.0 fM | 3 |
| Amp | 20.0 fm-10.0 pM | 10.0 fM | 4 |
| TENG | 10^-12^ -10^-7^ M | 0.1 pM | This work |

**Figure S1.** XPS spectrum of PDMS film.


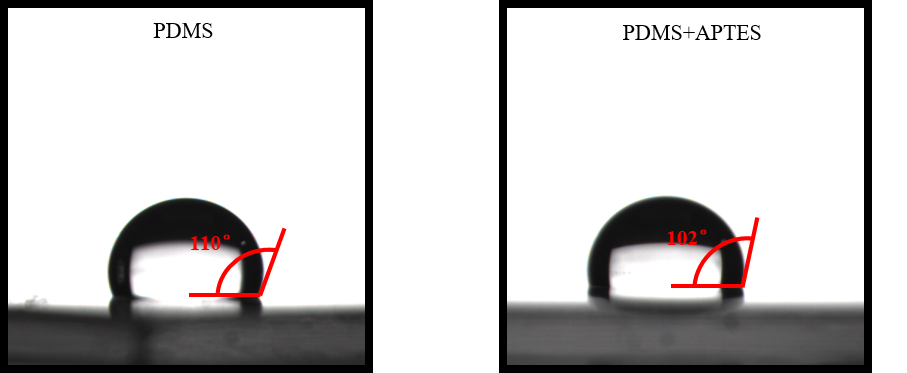


| （a）PDMS | （b）PDMS+APTES |
| --- | --- |


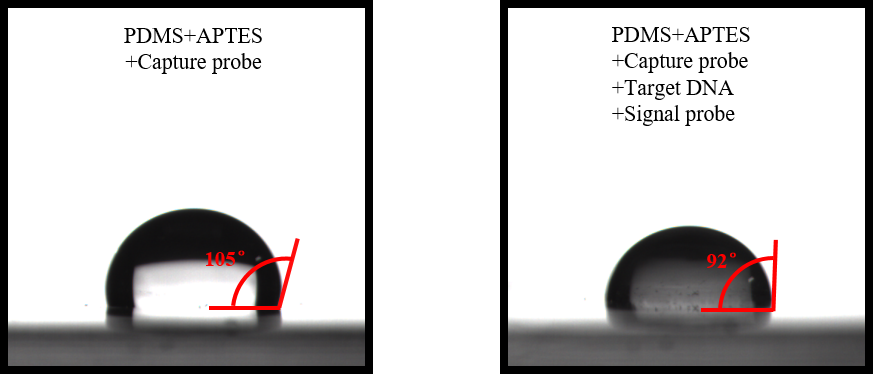


| （c）PDMS+APTES+Capture probe | （d）PDMS+APTES+Capture probe+Signal probe |
| --- | --- |

**Figure S2.** The contact angle of water on the PDMS film with different surface conditions.

**
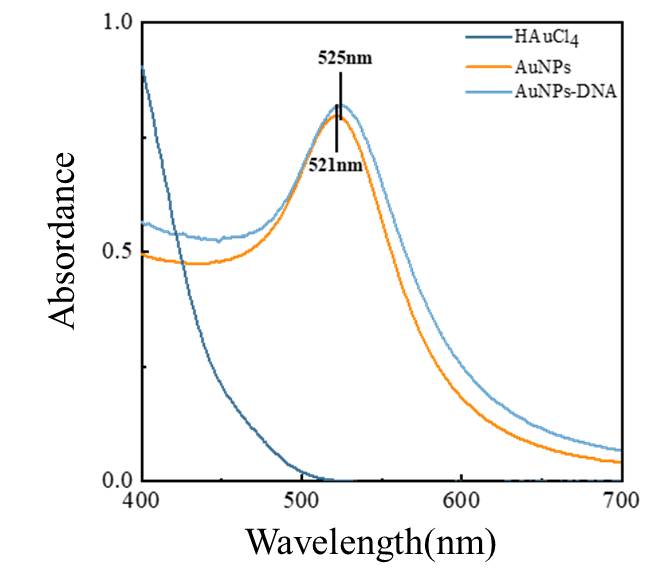
**

**Figure S3.** The UV-vis spectrum of AuNPs and DNA.


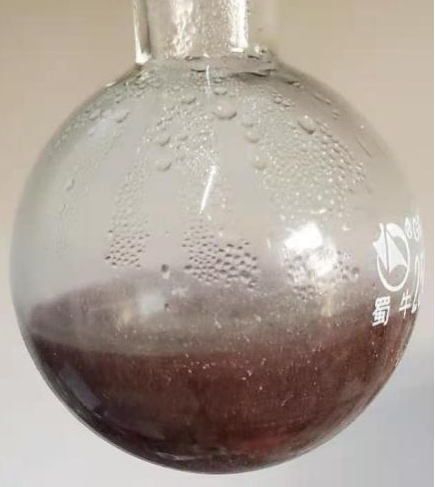


**Figure S4.** Color of AuNPs.


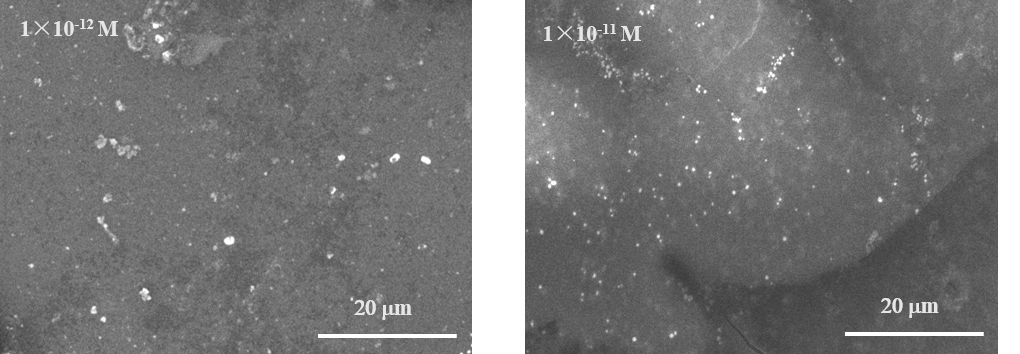


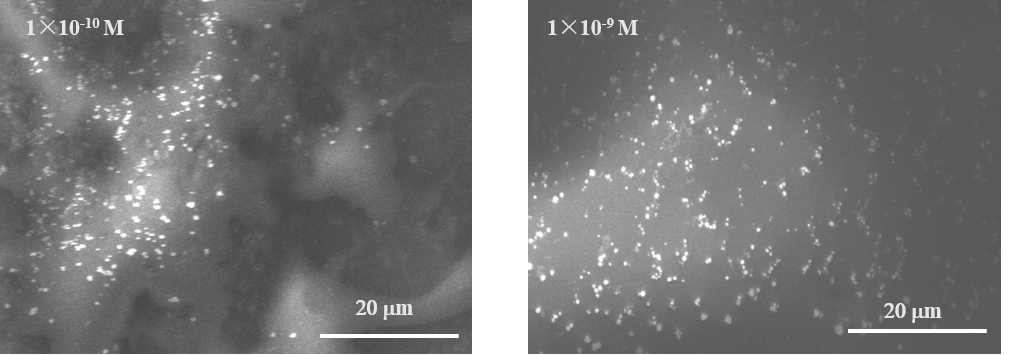


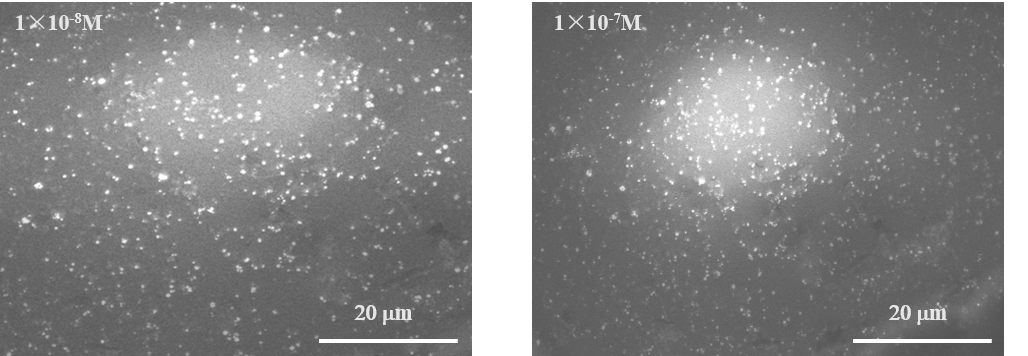


**Figure S5.** The UV-vis spectrum of AuNPs and DNA.


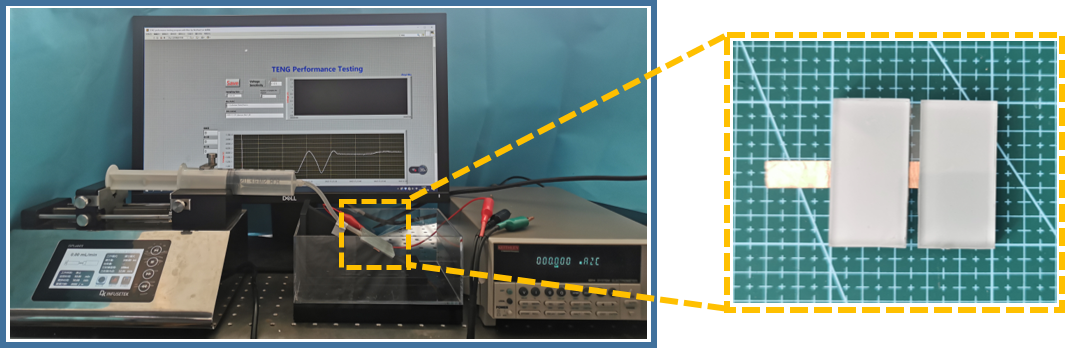


**Figure S6.** Experimental rig of liquid-solid TENG-based DNA barcode detection sensor.


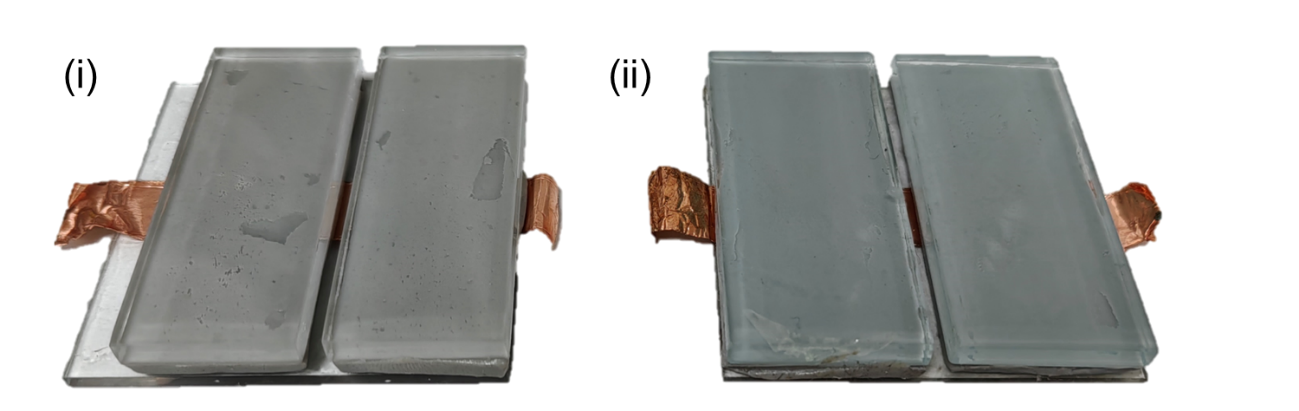


**Figure S7**. Different batches of TENG-based sensor made in (i) October and (ii) July.


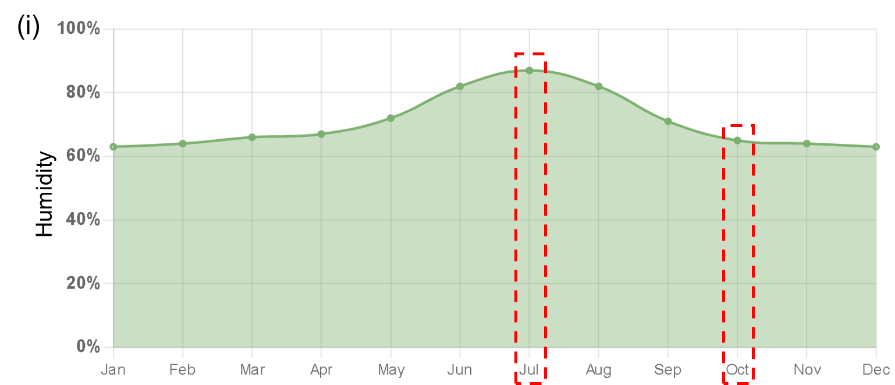


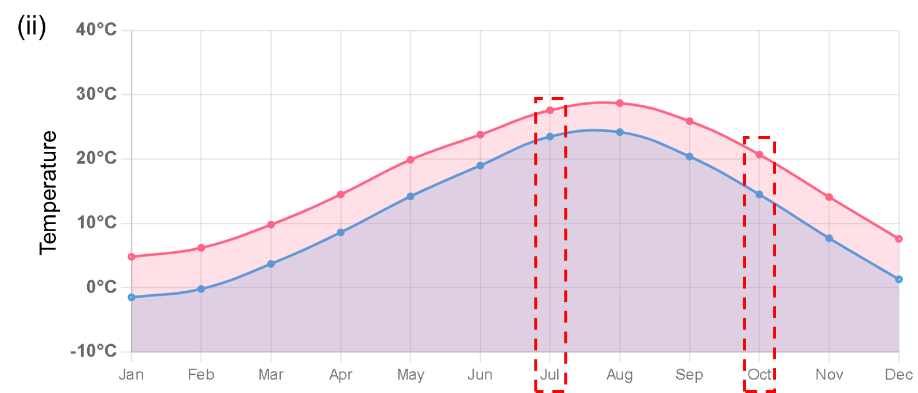


**Figure S8**. Weather parameters for different months in Qingdao. (i) average humidity (ii) average temperature, red line represents day temperature, blue line represents night temperature.

**Figure S9**. Typical sensor signal for different batches and time.

**Figure S10**. Typical sensor signal for different DNA barcode for various shrimp species.

**Figure S11.** Typical sensor signal for different DNA barcode for various shrimp species.

**Figure S12**. Typical sensor signal for different DNA barcode for the new batch of TENG-based biosensor.

**Figure S13**. Typical liquid-solid TENG signal for mismatched target DNA sequences.


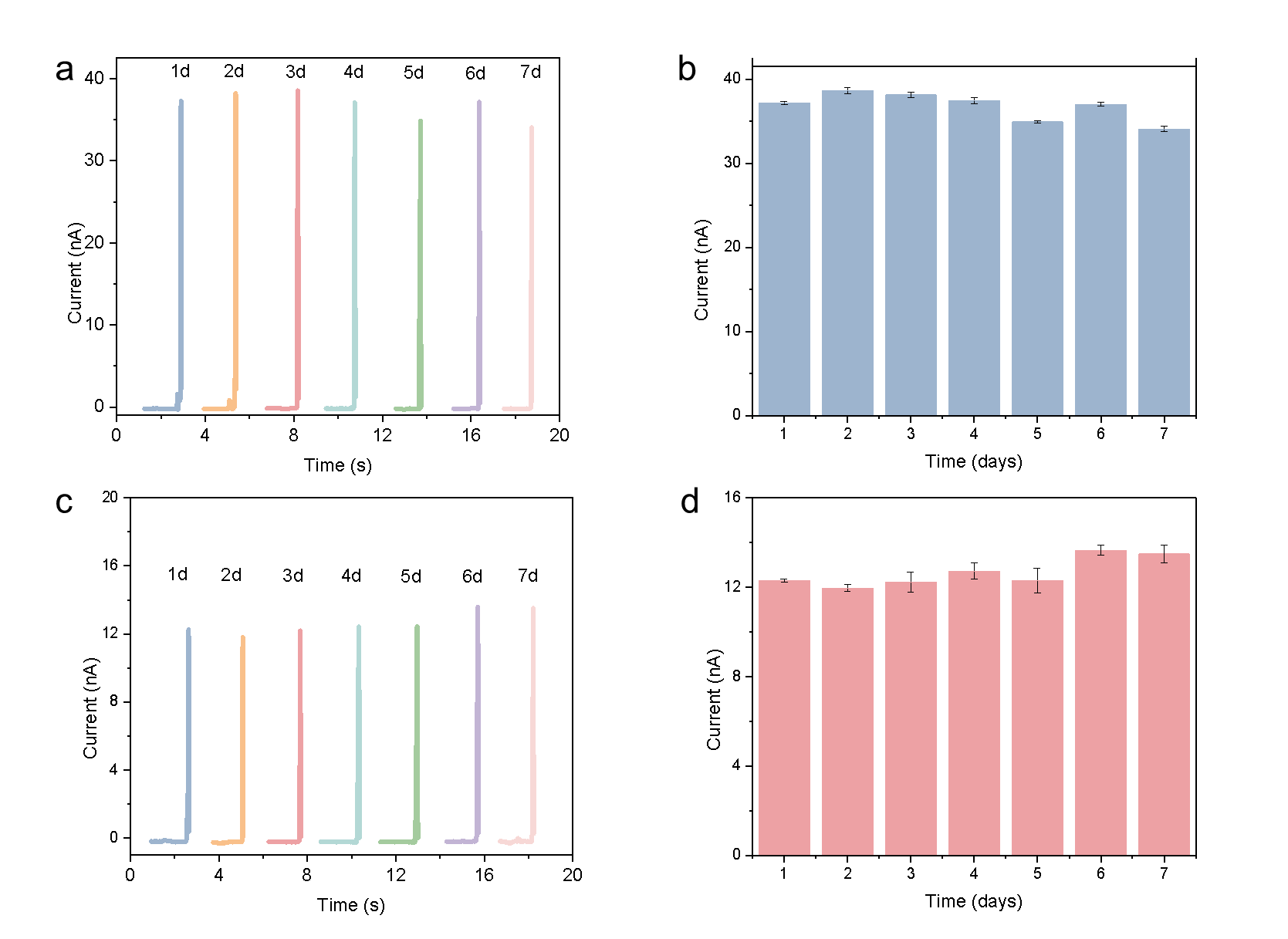


**Figure S14**. Long-term stability test of liquid-solid TENG-based DNA biosensor. (a) Typical current signal for blank group at different days; (b) Average current for blank group at different days; (c) Typical current signal for target DNA at different days; (d) Average current for target DNA at different days

**NoteS1: *Synthesis of AuNPs***

100 mL of deionized water and 2 mL of 1% HAuCl_4_ were added to a 250 mL round-bottom flask, which was sequentially stirred and heated in 95°C water bath. Then, 5 mL of 1% sodium citrate was added, followed with continual heating and stirring for 30 min. The color of the solution changed from light yellow to black, and finally to wine red. Figure S4 shows the final solution color, which is consistent with the phenomenon in the literature^[1]^. After the solution naturally cooled to room temperature, the product was filtered through a 200 nm filter membrane to collect the prepared gold nanoparticles and placed in a 4°C refrigerator for storage.

**NoteS2: *Synthesis of Signal Probe***

The signal probe is a DNA fragment modified by thiol groups. The thiol-modified DNA was dissolved to 1 μM using TE buffer solution (10 mmol/L Tris.HCl; 1 mmol/L EDTA). The signal probe was connected to AuNPs using the method in the literature^[2,3]^. ​​The thiol-modified DNA was purified by standard desalting method. 200 μl of 1 μM thiol-modified DNA was mixed with 5 μl of 0.1 M TCEP, and kept at room temperature for 30 min. Followingly, it was directly mixed with the AuNPs solution, and kept overnight. To remove excess oligomer sequences, the prepared DNA-Au NPs need to be centrifuged at 12,000 rpm for 10 min, resuspended three times with 1×PBS buffer solution, and finally dispersed in 1×PBS buffer solution.

**NoteS3: *Comparison between TENG-based biosensors and other sensors***

As far as the specificity is concerned, the linear range and LOD of present TENG-based biosensor are 10^-12^ -10^-7^ M and 0.1 pM, respectively. As shown in the Table S2, the specificity of the present biosensor is better than sensors based on DPV^[4]^ and CV^[5]^ designed in previous research, but worse than sensors transduced by EIS^[6]^ and Amp^[7]^. Besides, the cost of TENG-based biosensor can be approximately estimated by analysing its components. The cost glass slid for substrate and fluid channel, PDMS film and DNA capture probe is around $5. And manufacturing and self-designed signal collection circuit spend about $80. Therefore, the total cost of the TNEG-based sensor is just around $85. However, the common potentiostat for electrochemical detection of DNA is easily above $1000, which is much higher than our DNA detection rig.

**NoteS4: *Evaluation Test for reproducibility of sensors in different batches for various seasons***

Two batches of sensors (Figure S7) have been fabricated in July and October this year, which are applied to measure the DNA barcode of *Alvinocaris muricola* in different seasons. As shown in Figure S8, the average humidity and temperature for July are 87% and 27.6℃, however, the above parameters for October are 65% and 20.7℃. Consequently, the measured data for the above working conditions can be applied to address the effect of potential source or errors on the sensor performance in the actual application process.

The typical signal measured by different batches of sensors on different months is shown in Figure S9. It can be seen that, on both July and October, the current signal measured by sensor with attachment of *Alvinocaris muricola* DNA-barcode is evidently lower than the original current signal for sensor without attachment of target DNA, suggesting that the liquid-solid TENG-based sensor in present research still can successfully detect the target DNA, regardless of various batches and environmental conditions. As for the slight discrepancy of current signal on July and October, it can be attributed to the difference of humidity. The moisture in atmosphere can easily adsorb on the friction layer interface and form a thin water layer, accelerating the dissipation process of triboelectric charges. Therefore, high humidity leads to a relatively low current signal. However, the evident discrepancy between current signal without and with target DNA still can be successfully applied for DNA barcode detection.

**NoteS5: *Evaluation test of TENG-based sensor for other species and complicated biological samples***

As far as the other species is concerned, the DNA-barcode of *Rimicaris chacei* is chosen as the new target DNA, and novel capture probe was designed. According to the same fabrication and measurement process, new type of TENG-based sensor for detecting *Rimicaris chacei* (other species of shrimps) is applied to distinguish the DNA-barcode of *Alvinocaris muricola* (previous target DNA), *Rimicaris chacei* (new target DNA), *Chorocaris paulexa*, *Rimicaris sp. C39*. As shown in Figure S10, the current signal for *Rimicaris chacei* is low, which is similar to the signal for previous targe DNA (*Alvinocaris muricola*). However, the current signal for DNA barcode of other species is high. Consequently, it can be concluded that the TENG-based sensor can also be applied to distinguish the other species.

To evaluate the performance of a TENG-based sensor for complex biological samples, the mixture of target DNA (*Alvinocaris muricola*) and interfering DNA (including *Rimicaris chacei*, *Chorocaris paulexa*, and *Rimicaris sp. C39*) are applied to measure the output current signal, as shown in Figure S11. It can be seen that the TENG-based sensor current signal for target DNA mixed with other DNA is still evidently lower than that for the blank group, suggesting that the novel sensor in the present research can accurately detect the target DNA, regardless of the interfering effect of other DNA.

**NoteS6: *Evaluation test of stability of TENG-based biosensor***

To evaluate the long-term stability of the liquid-solid TENG-based DNA biosensor, we have measured the current signal for blank group and target DNA at different days. As shown in the Figure S14a and S14b, there exists an evident current spike signal for blank group at different days, and the peak value of current signal keeps stable. As far as the target DNA is concerned, the output current signal is lower than that for blank group at different days, suggesting that the liquid-solid TENG-based biosensor can successfully identify the target DNA (Figure S14c). Additionally, the peak value of current signal for target DNA keeps stable as well. Above all, it can be concluded that the liquid-solid TENG-based biosensors has a good stability.

*Refernces:*

1. Wang W, Ding X, Xu Q, Wang J, Wang L, Lou X. Zeta-potential data reliability of gold nanoparticle biomolecular conjugates and its application in sensitive quantification of surface absorbed protein. Colloids and Surfaces B: Biointerfaces. 2016 Dec 1;148:541-8.

2. Liu J, Lu Y. Colorimetric Cu 2+ detection with a ligation DNAzyme and nanoparticles. Chemical communications. 2007(46):4872-4.

3. Cordray MS, Amdahl M, Richards-Kortum RR. Gold nanoparticle aggregation for quantification of oligonucleotides: optimization and increased dynamic range. Analytical biochemistry. 2012 Dec 15;431(2):99-105.

4. Bettazzi F, Hamid-Asl E, Esposito CL, Quintavalle C, Formisano N, Laschi S, Catuogno S, Iaboni M, Marrazza G, Mascini M, Cerchia L. Electrochemical detection of miRNA-222 by use of a magnetic bead-based bioassay. Analytical and bioanalytical chemistry. 2013 Jan;405:1025-34.

5. Cai Z, Song Y, Wu Y, Zhu Z, Yang CJ, Chen X. An electrochemical sensor based on label-free functional allosteric molecular beacons for detection target DNA/miRNA. Biosensors and Bioelectronics. 2013 Mar 15;41:783-8.

6. Ren Y, Deng H, Shen W, Gao Z. A highly sensitive and selective electrochemical biosensor for direct detection of microRNAs in serum. Analytical chemistry. 2013 May 7;85(9):4784-9.

7. Gao Z, Peng Y. A highly sensitive and specific biosensor for ligation-and PCR-free detection of MicroRNAs. Biosensors and Bioelectronics. 2011 May 15;26(9):3768-73.
